# 

**Published:** 1846-12

**Authors:** 


*Single numbers of the Journal.*—We are sometimes applied to to furnish single numbers of the Journal. As we publish a limited edition it is important not to break sets, and we are therefore compelled to decline such requests. Contributors of articles will receive, if they desire it, additional copies of their own communications; and any number of copies can be struck off and published in a pamphlet form, at a small expense.

Several Bibliographical Notices, prepared for this Number, and in the hands of the compositor, are crowded out. Books which have been received will be severally noticed as time and space permit. We beg to repeat our general acknowledgment for numerous favors. Among others we have received a paper devoted to the Natural History of the Alligator, by Dr. Dowler, of New Orleans, written in his quaint, sprightly style, and conveying much interesting and instructive information respecting the organization and habits of the American Crocodile. We would also mention an Address by Dr. Stille, of Philadelphia, on Medical Education, which we have read with much pleasure.

